# Rifampicin-induced challenges in managing endocrine hypertension and primary aldosteronism: a case report and literature review

**DOI:** 10.3389/fphar.2025.1678430

**Published:** 2025-11-07

**Authors:** Xiaoxiao Song, Minyue Jia, Hanxiao Yu, Zhichao Dong, Kai Cheok, Xin Pan

**Affiliations:** 1 Department of Endocrinology, The Second Affiliated Hospital, Zhejiang University School of Medicine, Hangzhou, Zhejiang, China; 2 Department of Ultrasonography, The Second Affiliated Hospital, Zhejiang University School of Medicine, Hangzhou, Zhejiang, China; 3 Clinical Research Center, The Second Affiliated Hospital, Zhejiang University School of Medicine, Hangzhou, Zhejiang, China; 4 Department of Urology, The Second Affiliated Hospital, Zhejiang University School of Medicine, Hangzhou, Zhejiang, China; 5 Department of Endocrinology, The First People’s Hospital of Xiaoshan District, Hangzhou, Zhejiang, China

**Keywords:** primary aldosteronism, resistant hypertension, endocrine hypertension, drug-drug interactions, rifampicin

## Abstract

**Background:**

Primary Aldosteronism (PA), a form of endocrine hypertension (EH), often manifests as Resistant Hypertension (RHTN). RHTN is an increasingly prevalent clinical condition associated with target organ damage and a poor prognosis. Accurate diagnosis and management of EH and PA are challenging due to their diverse clinical manifestations, complex laboratory findings, and potential drug-drug interactions (DDIs). These DDIs, often overlooked in practice, can complicate the diagnostic and treatment processes.

**Case Presentation:**

A 56-year-old man with uncontrolled hypertension was admitted to our hospital. He was suspected of having Primary Aldosteronism (PA) and subclinical Cushing’s Syndrome (SCS) based on elevated aldosterone-to-renin ratio (ARR), captopril challenge test results (CCT), and low-dose dexamethasone suppression test (LDDST) results. Adrenal CT showed mild bilateral adrenal hyperplasia. Despite being on six antihypertensive medications, including spironolactone, his blood pressure remained uncontrollable. His medical history revealed prior use of rifampicin for brucellosis. Rifampicin, a CYP450 inducer, caused drug-drug interactions (DDIs), leading to a false-positive dexamethasone suppression test (DST) and reduced efficacy of antihypertensive drugs. After discontinuing rifampicin, his blood pressure was controlled with fewer medications. One month later, repeated ARR and CCT were still positive. Adrenal venous sampling (AVS) indicated bilateral aldosterone secretion without a dominant side, confirming Idiopathic Hyperaldosteronism (IHA). Targeted treatment with MRA led to partial clinical and biochemical remission of PA.

**Conclusion:**

This case highlights the diagnostic and therapeutic challenges of Endocrine Hypertension (EH) and Primary Aldosteronism complicated by CYP450 enzyme inducers. Specifically, the use of rifampicin, a potent CYP450 inducer, resulted in false-positive diagnostic test results and diminished efficacy of antihypertensive medications, thereby contributing to RHTN. When encountering uncontrolled hypertension, particularly when standard treatments fail, awareness of DDIs is crucial for accurate diagnosis and effective management.

## Introduction

Resistant hypertension (RHTN), a severe form of hypertension, is more likely to cause target organ damage and has a poor prognosis. It includes Endocrine hypertension (EH), where elevated blood pressure (BP) is closely linked to hormone secretion, most commonly due to adrenal diseases such as Primary aldosteronism (PA). Additionally, numerous factors influence the diagnosis and treatment of EH, including drug-drug interactions (DDIs). Studies indicate that 10%–20% of hypertensive patients develop resistant hypertension (RHTN), and roughly 5% of these cases are classified as extreme refractory hypertension (Siddiqui and Calhoun, 2017). Prompt identification of the underlying cause can markedly reduce blood pressure or even cure the hypertension. Nevertheless, the differential diagnostic process is not always straightforward in clinical practice. Here, we report a rare case of primary aldosteronism (PA) presenting as RHTN, in which diagnosis and treatment were complicated by DDIs involving rifampicin.

## Case presentation

**FIGURE 1:**
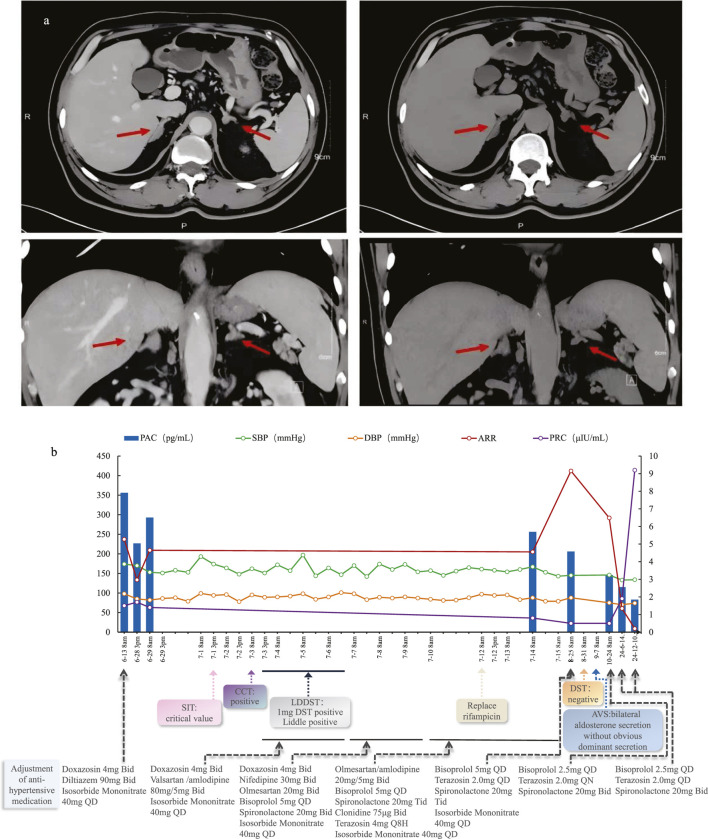
**(a)** Axial and Coronal adrenal contrast-enhanced CT of this patient; **(b)** Patient’s diagnostic and treatment process chart.

A 56-year-old Chinese man was admitted to the endocrinology department with suspected Primary aldosteronism (PA). He had an 18-year history of hypertension (regularly treated with nifedipine 30 mg QD), spontaneous hypokalemia and a history of cerebral infarction. The blood pressure (BP) had been significantly elevated for 1 month prior to hospitalization, with an admission BP of 174/98 mmHg. Physical examination revealed no Cushingoid features except for being overweight (BMI = 27.6 kg/m^2^). Laboratory tests revealed a positive aldosterone-to-renin ratio (ARR) due to an elevated plasma aldosterone concentration (PAC = 356.0 pg/mL; reference range: 30–353.0 pg/mL) and a decreased plasma renin concentration (PRC = 1.5µIU/mL; reference range 4.4–46.1μIU/mL). Cortisol levels at 8a.m. and 0a.m. (358.0 nmol/L and 145.0 nmol/L, respectively) suggested an abnormal circadian rhythm. Blood and urine catecholamine levels were within the normal range. Adrenal enhanced CT (Figure 1F1a) showed mild bilateral adrenal hyperplasia. Synthesizing the above situation, the patient was prescribed Doxazosin 4 mg BID and Diltiazem 90 mg BID for drug elution, and was also prescribed Isosorbide mononitrate 40 mg QD due to chest tightness. Two weeks later, he underwent a series of retests.

Routine laboratory tests revealed normal renal function and fasting blood glucose levels, and a negative renal artery ultrasound. An echocardiogram showed an ejection fraction (EF) of 67%, left atrial enlargement, and left ventricular hypertrophy. 24-h ambulatory BP monitoring Before admission indicated the disappearance of the BP circadian rhythm, with a non-dipper curve. His average systolic BP was 177 mmHg, peaking at 211 mmHg, while the average diastolic BP was 97 mmHg, peaking at 120 mmHg. Despite adequate potassium supplement, the serum potassium was only 3.16 mmol/L. A positive result (ARR = 209.29) was obtained again after a 2-week drug washout. Further confirmatory tests revealed that the saline suppression test (SIT) and captopril challenge test (CCT) showed all positive results, confirming PA. We re-evaluated his cortisol levels [(8a.m.-4p.m.-0a.m.) = 187.0-167.6-259.6 nmol/L, respectively; reference range: 166.0–507.0 nmol/L for 8a.m.] and adrenocorticotropic hormone (ACTH) levels [(8a.m.-4p.m.-0a.m.) = 61.3-46.2-66.5 pg/mL, respectively; reference range: 7.2–63.3 pg/mL for 8a.m.], which implied a disrupted ACTH-cortisol rhythm. The overnight 1-mg dexamethasone suppression test (ODST) was performed first, yielding a cortisol level of 113.0 nmol/L. Subsequently, the low-dose 2-day dexamethasone suppression test (LDDST) resulted in a cortisol level of 193 nmol/L. These findings suggested the possibility of endogenous hypercortisolism.

## Diagnosis and treatment process

Given the above situation, we considered the following preliminary diagnoses: Endocrine hypertension (EH): Primary aldosteronism (PA), possible Cushing’s syndrome (CS). Although spironolactone (20 mg TID) and amlodipine (5 mg BID) were added for the treatment of PA, there was no significant change in systolic BP. The BP occasionally reached 200/100 mmHg. Subsequent treatment involved bisoprolol (5 mg QD), terazosin (4 mg Q8H), clonidine (75 μg BID), and Olmesartan (20 mg BID) at near-maximum doses, but hypertension remained uncontrolled, indicating the presence of RHTN.

Considering the ACTH levels and positive LDDST results, we initially suspected that Cushing’s Syndrome (CS) was causing the resistant hypertension. However, a pituitary MRI yielded negative findings. Then, after a multidisciplinary team (MDT) discussion and a review of the patient’s medical history, it was revealed that he had been regularly taking rifampicin (600 mg QD) combined with doxycycline (100 mg BID) for brucellosis 1 month before admission. The patient did not disclose this history initially, as he believed that the previous treatment for brucellosis was irrelevant to the current hospitalization. Moreover, upon further reflection, he confirmed that his blood pressure (BP) became uncontrollable after 10 days of brucellosis treatment. Given that rifampicin acts as a hepatic microsomal CYP450 enzyme inducer and can lead to drug-drug interactions (DDIs), we decided to replace it with sulfamethoxazole (SMZ) (Qureshi et al., 2023). Three days after changing from rifampicin to SMZ, the diurnal BP fluctuations improved significantly. This further confirmed that the patient’s uncontrollable BP, despite the use of multiple antihypertensive agents, was associated with the decreased plasma concentrations of some antihypertensive drugs caused by rifampicin, indicating the occurrence of DDIs in this patient’s treatment process. We recommended that the patient undergo follow-up and re-examination in the outpatient clinic 1 month later.

## Follow-up and clinical outcome

Two weeks after discharge, the patient’s BP decreased. His antihypertensive regimen had been reduced to spironolactone (20 mg TID), bisoprolol (5 mg QD), terazosin (2.0 mg QD), isosorbide mononitrate (40 mg QD), and potassium supplementation 1.0 g per day. Approximately 1 month after discharge, he underwent an outpatient follow-up, 1 month after completing the brucellosis treatment course. Laboratory tests revealed a plasma aldosterone concentration (PAC) of 206 pg/mL and a positive ARR. The overnight DST was repeated, yielding a negative result (cortisol = 17.6 nmol/L). The previous LDDST results were attributed to rifampicin use and considered falsely positive. The patient was diagnosed with PA but not CS. Adrenal venous sampling (AVS) indicated bilateral aldosterone secretion without a dominant side. The patient was ultimately diagnosed with idiopathic hyperaldosteronism (IHA) and elected to undergo long-term MRA centered medical therapy.

Three months after discharge, his antihypertensive medications had already been reduced to spironolactone (20 mg BID), bisoprolol (2.5 mg QD), and terazosin (2 mg QD). This antihypertensive regimen remained unchanged until the 1.5-year follow-up after discharge when he returned for another visit. During the 1.5-year follow-up, his PAC was 83.0 pg/mL, PRC was 9.2 µU/mL, and ARR was 9.02. Throughout the entire post-discharge follow-up period, both his blood pressure and potassium levels remained normal. The diagnosis and treatment process is shown in Figure 1F1b.

## Discussion and Conclusion

Primary aldosteronism (PA) is the most common cause of endocrine hypertension (EH) and can manifest as resistant hypertension (RHTN). However, some cases of PA are actually complicated by the presence of pseudo-RHTN, which can result from CYP450 enzyme inducers that interfere with diagnostic tests and treatment due to drug-drug interactions (DDIs).

Pharmacokinetic-based DDIs are most common and involve various enzymes, with the cytochrome P450 (CYP450) family being the most critical for human metabolism (Michalets, 1998). As a CYP450 inducer, rifampicin increases drug bioconversion rates and lowers serum drug concentrations, which may reduce efficacy or cause treatment failure. Studies show that the peak induction effect of rifampicin occurs at a daily dose of 600 mg, with higher doses failing to significantly enhance DDIs (Gorski et al., 2003). In this patient, PA was confirmed by hypertension, spontaneous hypokalemia, a positive ARR, and confirmatory CCT. However, the differential diagnosis and treatment of endocrine hypertension were complicated by the use of rifampicin.

CS is a clinical syndrome caused by prolonged excessive cortisol secretion from the adrenal cortex. Some patients with PA can also have concurrent CS. This patient lacked typical CS features but had abnormal initial hormone assays of DST, raising suspicion for subclinical Cushing’s syndrome (SCS). Studies show that approximately 21% of PA patients may have concurrent SCS (Hiraishi et al., 2011). Initially, we hypothesized that the patient’s uncontrollable hypertension was due to combined PA and SCS. However, upon identifying rifampicin use, the dramatic BP fluctuations and false-positive LDDST results were clearly attributed to DDIs.

Drug-drug interactions (DDIs) occur when the pharmacokinetics or pharmacodynamics of a drug are altered by the presence of another drug, potentially leading to adverse effects or changes in therapeutic efficacy. These interactions can be influenced by factors such as drug-metabolizing enzymes, like CYP450, and drug transport proteins (Subr et al.). Rifampicin, an inducer of hepatic microsomal CYP450 enzymes including CYP3A4, CYP2C19, CYP2B6, CYP2C8, and CYP2C9 et al., accelerates the metabolism of various drugs like glucocorticoids, warfarin, and oral contraceptives that are metabolized by the CYP450 enzyme family (Baciewicz and Self, 1984). Among these, CYP3A4 is involved in the metabolism of a large proportion (30%–50%) of clinically used drugs (Liu et al., 2005; Haddad et al., 2007). The enzyme activity changes induced by rifampicin peak 1–2 weeks after oral administration (Bettonte et al., 2023). By inducing CYP3A4 activity, rifampicin can increase the clearance of dexamethasone fivefold and decrease its half-life threefold (Kawai, 1985; Evans et al., 1985). This results in reduced dexamethasone concentrations, which fail to suppress endogenous cortisol, leading to the aforementioned false-positive results in the DST (Chinese Pituitar y Adenoma Collaboration Group, 2016).

Additionally, the biosynthesis and catabolic metabolism of cortisol, aldosterone, and sex steroid hormones also involve multiple enzymes within the CYP450 family. Rifampicin enhances cortisol catabolism, thereby reducing plasma cortisol concentrations. This reduction in cortisol levels leads to elevated ACTH levels, which in turn stimulate an increased rate of cortisol synthesis (Edwards et al., 1974; Elias and Gwinup, 1980). The impact of rifampicin on the metabolism of aldosterone and catecholamines remains to be fully elucidated. However, it has been demonstrated that rifampicin can interfere with high-performance liquid chromatography (HPLC) measurements of urinary catecholamines, resulting in falsely elevated results (Kim et al., 2015).

Furthermore, most antihypertensive drugs are substrates of CYP450 enzymes. Since rifampicin can induce these enzymes, it leads to decreased blood concentrations and reduced efficacy of the antihypertensive medications (see Table 1), thereby causing elevated blood pressure. Consequently, more targeted selection of antihypertensive drugs is required for patients receiving concurrent treatment with rifampicin. Among the antihypertensive drugs available at our hospital, we selected olmesartan (which is not affected by CYP450), amlodipine (which is relatively less affected), and terazosin (for which no definitive evidence of interaction has been observed) for this patient’s subsequent antihypertensive treatment. Although the efficacy of bisoprolol was affected by rifampicin’s CYP enzyme induction, it was still retained and continued to be used to alleviate sympathetic excitation. In this patient’s case, during the period when he was using rifampicin, his antihypertensive regimen was gradually increased to six medications (even with concurrent treatment for PA with mineralocorticoid receptor antagonists such as spironolactone). Despite this, all medications were administered at near-maximal doses, yet his hypertension remained poorly controlled.

**TABLE 1:** The Mechanism by which Rifampicin Affects the Efficacy of Some Antihypertensive Drugs.

Drug category	Drug name	Antihypertensive efficacy	The effect of rifampicin on the CYP450 enzyme system			
			CYP3A4	CYP2C19	CYP2C9	CYP2D6
CCB	Nifedipine (Yoshimoto et al., 1996)	↓	activity↑	-	-	-
	Amlodipine (Ye et al., 2019)	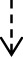	activity↑	-	-	-
	Verapamil (Barbarash, 1985)	↓	activity↑	-	-	-
	Diltiazem (Adebayo et al., 1989)	↓	activity↑	-	-	-
Beta blocker	Metoprolol (Molden and Spigset, 2011)	↓	-	-	-	activity↑
	Propranolol (McGinnity et al., 2000)	↓	-	-	-	activity↑
	Bisoprolol (Horikiri et al., 1998)	↓	activity↑	-	-	activity↑
ACEI	Captopril (Gan et al., 2014)	-	-	-	-	-
ARB	Losartan (Williamson et al., 1998; Joy et al., 2009)	↓	activity↑	-	activity↑	-
	Valsartan (Marino and Vachharajani, 2001)	↓	-	-	activity↑	-
	Olmesartan (Laeis et al., 2001)	-	-	-	-	-
Alpha blocker	Prazosin (Akduman and Crawford, 2001)	-	-	-	-	-
	Terazosin	—	—	—	—	—
Diuretic	Indapamide (Yan et al., 2012; Sun et al., 2009)	↓	activity↑	activity↑	-	-
	Furosemide (Gan et al., 2014)	-	-	-	-	-
	Hydrochlorothiazide (Gan et al., 2014)	-	-	-	-	-
	Active metabolites of Spironolactone (Soliman et al., 2025; Niwa et al., 2020)	↓	activity↑	-	-	-

**CCB: calcium channel blockers; ACEI: angiotensin converting enzyme inhibitors; ARB: angiotensin receptor blockers; -: no effect;/: not reported;: The dashed line indicates that amlodipine, which is both metabolized by and acts as an inhibitor of CYP3A4, partially counteracts the CYP3A4 activation induced by rifampicin.

Spironolactone is a prodrug extensively metabolized in the liver to active metabolites, mainly 7α-thiomethyl-spironolactone (7α-TMS) and canrenone, of which 7α-TMS shows greater and more sustained mineralocorticoid receptor antagonism than the parent drug (Soliman et al., 2025; Overdiek and Merkus, 1987; ALDACTONE, 2008). Although spironolactone itself is not primarily metabolized by cytochrome P450 enzymes, 7α-TMS may undergo CYP3A4-mediated hydroxylation to form the less active 6β-hydroxy-7α-TMS (Soliman et al., 2025; Niwa et al., 2020; ALDACTONE, 2008; Flowers et al., 1989). This oxidative pathway regulates the duration of spironolactone’s pharmacologic effect, and rifampicin-induced CYP3A4 upregulation may accelerate 7α-TMS clearance, thereby reducing therapeutic efficacy (Soliman et al., 2025; Niwa et al., 2020).

After replacing rifampicin with sulfamethoxazole for the subsequent brucellosis treatment, blood pressure variability significantly improved (Figure 1b). Additionally, the enzymatic activity changes induced by rifampicin typically disappear about 2 weeks after discontinuation (Niemi et al., 2003; Imai et al., 2011). Although the exact time required for rifampicin elution is not definitively established, some scholars recommend performing DST 15 days after discontinuation (Abdullah and Nowalid, 2010). After rechecking DST and ARR levels 1 month later, a true negative DST outcome was observed, while both the ARR and CTT values remained elevated. These results ultimately led to a diagnosis of idiopathic hyperaldosteronism (IHA) for the patient, with CS ruled out.

During the follow-up phase of treatment, his hypokalemia was corrected and PRC increased to nearly 10µIU/mL. According to the Primary Aldosteronism Medical Treatment Outcome (PAMO) criteria (Yang et al., 2025), the patient achieved a partial clinical and biochemical response. Additionally, using the defined daily dose (DDD) as a standard and following the DDD analysis method recommended by WHO Collaborating Center (WHOCC, 2017), we calculated the antihypertensive drug consumption of the patient on the day of discharge and at the last follow-up visit. Compared to the period when he was using rifampicin, the total accumulated DDD of antihypertensive medications (excluding the targeted medication-spironolactone) decreased by over 50% (Table 2).

**TABLE 2:** Total accumulated DDD copies of the patient’s antihypertensive medications.

Drug name	DDD (mg)	Initial antihypertensives (with rifampicin)		Current antihypertensives (without rifampicin)	
		Dosage and Usage	Accumulated DDD copies	Dosage and Usage	Accumulated DDD copies
Spironolactone	37.5	20 mg TID	1.6	20 mg BID	1.07
Terazosin	5	4 mg Q8H	2.4	2 mg QD	0.4
Bisoprolol	10	5 mg QD	0.5	2.5 mg QD	0.25
Amlodipine	5	5 mg BID	2		
Olmesartan	20	20 mg BID	2		
Clonidine	0.45	75 μg BID	0.33		
Total accumulated DDD copies (excluding spironolactone)			7.23		0.65

**FIGURE 2:**
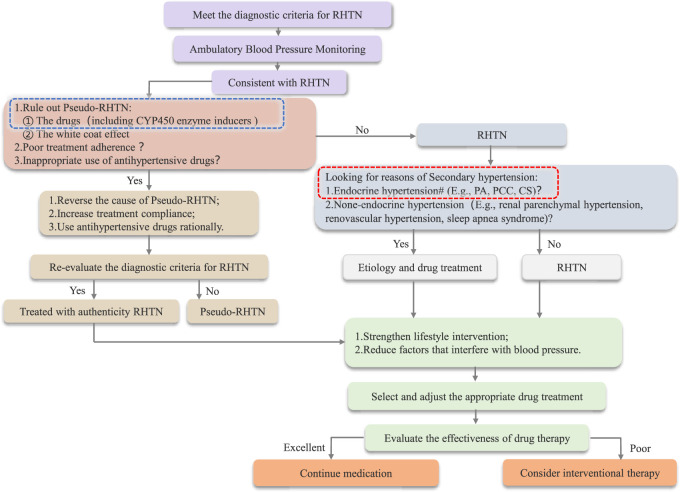
Flowchart Outlining the Diagnosis and Treatment of RHTN *: Drugs that cause DDIs by inducing abnormal metabolic activity of CYP450 enzymes can be categorized into two groups: CYP450 enzyme inducers and inhibitors. #: CYP450 enzyme disruptors can affect endocrine function test results; therefore, it is essential to ensure the proper elution of such disruptors.

Through this study, we optimized the diagnostic and therapeutic algorithm for resistant hypertension (RHTN), with particular emphasis on the role of CYP enzyme disruptors (Figure 2). Additionally, after reviewing relevant literature, we have compiled a reference list of common CYP450 enzyme inducers and their effects on CYP enzymes, including those that reduce antihypertensive drug efficacy and cause blood pressure fluctuations through enzyme induction (Table 3).

**TABLE 3:** Main drugs acting as CYP 450 enzyme inducers.

Enzyme											
Class of inducers	Inducing medication	CYP1A1	CYP1A2	CYP2A6	CYP2B6	CYP2C8	CYP2C9	CYP2C19	CYP3A4	CYP3A5	CYP3A7and CYP3A43
Antibiotic	Rifampicin		W (Hakkola et al., 2020)		M (Hakkola et al., 2020)	M (Stout et al., 2021)	M^a^	M^a^	S (Hakkola et al., 2020)	S^a^	ND
	Dicloxacillin						W (Hakkola et al., 2020)	W (Hakkola et al., 2020)	W (Hakkola et al., 2020)		
Antiepileptics	Carbamazepine		W (Magnusson et al., 2008)	W (Hakkola et al., 2020)	M (Hakkola et al., 2020)	ND	M^a^	M (Hakkola et al., 2020)	S^a^		ND
	Phenytoin		ND		ND		M (Rodriguez-Vera et al., 2023)	M (Rodriguez-Vera et al., 2023)	M (Hakkola et al., 2020)		
Proton pump inhibitors	Omeprazole	ND	W^a^								
Glucocorticoids	Dexamethasone								W^a^		
	Methylprednisolone								W (Hakkola et al., 2020)		
	Prednisolone								W (Hakkola et al., 2020)		
	Prednisone								Min. (Belderbos et al., 2019)		
Steroidogenesis inhibitors	Mitotane								S (Hakkola et al., 2020)		
Stimulants	Modafinil (and its Renantiomer armodafinil)								W^a^		
	Nelfinavir		M (Hakkola et al., 2020)		W (Hakkola et al., 2020)		W (Hakkola et al., 2020)				
Antiretrovirals	Ritonavir		M (Hakkola et al., 2020)		W-M (Hakkola et al., 2020)		Min. (Rohr et al., 2024)	M (Rohr et al., 2024)	M (Rohr et al., 2024)		
	Tipranavir								ND		
	Pentobarbital		ND				M (Hakkola et al., 2020)	ND	M (Hakkola et al., 2020)		
Barbiturates	Phenobarbital		W (Hakkola et al., 2020)	ND	S (Li et al., 2019)		W^a^	W (Hakkola et al., 2020)	M^a^		
	Secobarbital		ND				ND				
Antimalarials	Artemisinin			M (Burk et al., 2012)	M (Hakkola et al., 2020)			W (Hakkola et al., 2020)	M (Hakkola et al., 2020)		
Estrogens	Ethinyl estradiol (of oral contraceptives)			W (Benowitz et al., 2006)							
Antiandrogens	Apalutamide						S (Hakkola et al., 2020)	W (Hakkola et al., 2020)	M (Hakkola et al., 2020)		
	Enzalutamide						M (Hakkola et al., 2020)	M (Hakkola et al., 2020)	M (Hakkola et al., 2020)		
Antipyretic analgesic	Metamizole				M (Hakkola et al., 2020)				M (Bachmann et al., 2021)		
Gout medications	Lesinurad								W (G et al., 2017)		
Antiemetics	Aprepitant						W (Hakkola et al., 2020)		W (Shadle et al., 2004)		
Antidiarrheals	Telotristat ethyl								M (FDA, 2017)		
Antineoplastic agents	Vinblastine								W (Smith et al., 2010)		
Bile acid derivatives	Ursodeoxycholic acid								Min. (Dilger et al., 2005)		

CYP450 induction strength is indicated as follows.

S: strong effect; M: moderate effect; W: weak effect; Min.: minimal effect; ND: Not determined (An inducer, but public AUC/fold-change data are lacking for strength classification).

^a^ We thank Dr. David A. flockhart and the division of clinical pharmacology, Indiana University School of Medicine, for maintaining the Cytochrome P450 Drug Interaction Table (https://drug-interactions.medicine.iu.edu), which served as the key reference for the asterisked data in Table 3.

Beyond its potent CYP450 enzymes, rifampicin also upregulates intestinal and hepatic P-glycoprotein (P-gp/ABCB1) expression by approximately 3.5-fold in humans. P-gp is an ATP-dependent efflux transporter that actively pumps absorbed substrates back into the intestinal lumen, thereby reducing their net absorption and systemic bioavailability. Consequently, rifampicin primarily affects the oral exposure of P-gp substrates, including endocrine hormones and certain antihypertensive drugs, through intestinal P-gp induction (Greiner et al., 1999; Fromm, 2004).

Previous research has shown that aldosterone and cortisol are physiological but low-affinity substrates of P-gp (Ueda et al., 1992), whereas renin is not. In Caco-2 cell experiments, Crowe and Tan (2012) reported low efflux ratios (ER ≈ 1.5) for aldosterone and cortisol, indicating weak transport activity with limited clinical significance (Crowe and Tan, 2012). Therefore, rifampicin-induced upregulation of P-gp has minimal impact on plasma aldosterone levels, while renin remains unchanged, leaving the ARR largely unaffected. In contrast, dexamethasone is a well-established P-gp substrate (Caco-2 efflux ratio≈2.1) (Crowe and Tan, 2012). Rifampicin, through the combined induction of CYP450 enzymes and P-gp, markedly reduces oral dexamethasone exposure, which may result in false-positive outcomes during the DST.

On the other hand, across antihypertensive drug classes, the influence of P-gp on systemic exposure and therapeutic efficacy varies considerably. Among CCBs, pharmacogenetic studies indicate that amlodipine plasma levels are affected by *ABCB1* polymorphisms, suggesting modest P-gp involvement, though its disposition is largely governed by CYP3A metabolism (Kim et al., 2007). Nifedipine is also primarily metabolized by CYP3A and shows no evidence of being a P-gp substrate (Soldner et al., 1999). Diltiazem demonstrates a mild association with P-gp, as animal studies suggest that intestinal efflux may partially limit its absorption (Athukuri and Neerati, 2017), but it also acts as a P-gp inhibitor (Wessler et al., 2013). For β-adrenoceptor blockers, evidence of P-gp substrate activity is limited and varies by agent. Carvedilol appears to be the clinically relevant substrate and also a moderate inhibitor, whereas bisoprolol shows weak affinity, and other β-blockers such as metoprolol, atenolol, and sotalol exhibit negligible interaction (Wessler et al., 2013; Bachmakov et al., 2006). In the ARB and ACEI classes, olmesartan medoxomil exhibits intestinal absorption partly mediated by OATP2B1 at the prodrug stage. The active metabolite, olmesartan, is predominantly excreted into bile through MRP2, with limited evidence of direct involvement of P-gp (Fukazawa et al., 2024). For α_1_-adrenergic blockers such as terazosin and doxazosin, there is currently very limited evidence confirming P-gp transport. Among diuretics, spironolactone functions as a P-gp modulator rather than a substrate, primarily inducing P-gp expression through pregnane X receptor (PXR) activation *in vitro* studies (Rigalli et al., 2011).

Collectively, in this case of endocrine hypertension under rifampicin therapy, secondary induction of P-gp may contribute to clinically relevant effects such as false-positive results in the DST and mild, limited reductions in the efficacy of certain antihypertensive agents.

These combined pharmacokinetic mechanisms underscore the importance of careful diagnostic interpretation and therapeutic monitoring when potent enzyme or transporter inducers are used. Nevertheless, in this case, CYP450 enzyme induction remains the principal mechanism driving rifampicin-related reductions in antihypertensive drug exposure, whereas P-gp modulation serves as a confounding factor in these DDIs.

## Summary

This paper reviews a patient with Resistant Hypertension (RHTN) and Primary Aldosteronism (PA), highlighting the challenges in managing Endocrine Hypertension (EH) complicated by drug-drug interactions (DDIs). CYP450 enzyme inducers, particularly rifampicin, can interfere with EH diagnostic tests and alter antihypertensive drug metabolism, leading to treatment failure and persistent hypertension. These DDIs are often overlooked in RHTN. When laboratory results and clinical manifestations are discordant, potential influencing factors, including DDIs, should be carefully investigated. This approach can improve diagnostic accuracy and therapeutic outcomes, reducing the risk of misdiagnosis and inappropriate treatment.

## Data Availability

The original contributions presented in the study are included in the article/supplementary material, further inquiries can be directed to the corresponding authors.
